# PGI_2_ Analog Attenuates Salt-Induced Renal Injury through the Inhibition of Inflammation and Rac1-MR Activation

**DOI:** 10.3390/ijms21124433

**Published:** 2020-06-22

**Authors:** Daigoro Hirohama, Wakako Kawarazaki, Mitsuhiro Nishimoto, Nobuhiro Ayuzawa, Takeshi Marumo, Shigeru Shibata, Toshiro Fujita

**Affiliations:** 1 Division of Clinical Epigenetics, Research Center for Advanced Science and Technology, The University of Tokyo, Tokyo 153-8904, Japan; wkawarazaki-tky@umin.ac.jp (W.K.); nishimoto-tky@umin.ac.jp (M.N.); ayuzawa-tky@umin.ac.jp (N.A.); tmarumo-npr@umin.ac.jp (T.M.); shigeru.shibata@med.teikyo-u.ac.jp (S.S.); Toshiro.FUJITA@rcast.u-tokyo.ac.jp (T.F.); 2 Division of Nephrology, Department of Internal Medicine, Teikyo University School of Medicine, Tokyo 173-8606, Japan; 3 Department of Internal Medicine, International University of Health and Welfare Mita Hospital, Tokyo 108-8329, Japan; 4 Center for Basic Medical Research at Narita Campus, International University of Health and Welfare, Chiba 286-8686, Japan; 5 Shinshu University School of Medicine and Research Center for Social Systems, Nagano 389-0111, Japan

**Keywords:** blood pressure, inflammation, mineralocorticoid receptor, Rac1, renal injury, salt-sensitive hypertension

## Abstract

Renal inflammation is known to be involved in salt-induced renal damage, leading to end-stage renal disease. This study aims to evaluate the role of inflammation in anti-inflammatory and renoprotective effects of beraprost sodium (BPS), a prostaglandin I_2_ (PGI_2_) analog, in Dahl salt-sensitive (DS) rats. Five-week-old male DS rats were fed a normal-salt diet (0.5% NaCl), a high-salt diet (8% NaCl), or a high-salt diet plus BPS treatment for 3 weeks. BPS treatment could inhibit marked proteinuria and renal injury in salt-loaded DS rats with elevated blood pressure, accompanied by renal inflammation suppression. Notably, high salt increased renal expression of active Rac1, followed by increased Sgk1 expressions, a downstream molecule of mineralocorticoid receptor (MR) signal, indicating salt-induced activation of Rac1-MR pathway. However, BPS administration inhibited salt-induced Rac1-MR activation as well as renal inflammation and damage, suggesting that Rac1-MR pathway is involved in anti-inflammatory and renoprotective effects of PGI_2_. Based upon Rac1 activated by inflammation, moreover, BPS inhibited salt-induced activation of Rac1-MR pathway by renal inflammation suppression, resulting in the attenuation of renal damage in salt-loaded DS rats. Thus, BPS is efficacious for the treatment of salt-induced renal injury.

## 1. Introduction

Dietary high-salt intake does not only increase blood pressure but also induce renal injury. Accumulating evidence indicates that inflammation in the kidney plays a key role in salt-induced renal damage, leading to end-stage renal disease [1,2,3]. N-acetyl-seryl-aspartyl-lysyl-proline, a natural tetrapeptide with anti-inflammatory properties, prevented salt-induced renal damage without affecting the blood pressure in Dahl salt-sensitive (DS) rats [4]. However, it is still unknown how inflammation evoked by a high-salt diet leads to renal injury.

Rac1 is a member of the RhoGTPase subfamily that acts as an intracellular molecular switch, transducing extracellular stimuli to modulate multiple signaling pathways [5]. A signaling cross-talk between mineralocorticoid receptor (MR) and the small GTPase Rac1 as a novel pathway that modulates MR function have been previously identified [6,7,8]. We have demonstrated that salt loading increases Rac1 activity in the kidneys of DS rats, which is associated with MR activation and upregulation of the MR target gene serum and glucocorticoid-regulated kinase (Sgk1) expression despite reduced circulating levels of aldosterone, a ligand of MR, resulting in salt-induced kidney injury [7]. By contrast, Rac1 and MR activities were appropriately decreased by salt loading in Dahl salt-resistant (DR) and normotensive rats, which were not accompanied by kidney injury [7]. In addition, administration of Rac1 inhibitor NSC23766 suppressed MR activation and the kidney damage induced by salt loading, which was as effective as eplerenone treatment, an MR blocker [7]. Hence, Rac1-MR activation plays a key role in mediating salt-induced kidney injury in DS rats.

Beraprost sodium (BPS) is an orally available and chemically stable prostaglandin I_2_ (PGI_2_) analog with established safety. It is clinically used to treat pulmonary arterial hypertension [9] and peripheral arterial disease [10]. Several lines of evidence indicate that BPS has the renoprotective effect in various pathological conditions including anti-glomerular basement membrane (GBM) glomerulonephritis [11], obesity-related kidney damage [12], diabetic kidney disease [13,14], unilateral ureteral obstruction [15,16], and contrast-induced nephropathy [17], which is attributed to the anti-inflammatory effect of BPS [11,14,16,18]. BPS also improved survival rates in anti-GBM glomerulonephritis rats and 5/6 nephrectomized chronic kidney disease (CKD) rats [19]. However, the mechanism for the renoprotective effect of BPS is still unknown. Of note, high salt induces renal inflammation in salt-sensitive hypertensive rats [1,2,20], and PGI_2_ possesses anti-inflammatory action [11,14,16,18]. Moreover, based upon the previous reports indicating that inflammation activates Rac1 [21,22,23], there are some possibilities that BPS inhibits salt-induced Rac1-MR activation by inflammation inhibition, resulting in the attenuation of salt-induced renal damage.

These observations led us to the plausible hypothesis that BPS is therapeutically useful for the treatment of salt-induced renal damage through the suppression of activated Rac1-MR pathway. To test the hypothesis in this study, we investigated the renoprotective effect of BPS in the DS rat, which is a commonly used model of salt-sensitive hypertension [24].

## 2. Results

### 2.1. BPS Treatment Did Not Alter Blood Pressure Levels in High-Salt-Fed DS Rats

All animals completed the study protocol. Male DS rats received a normal-salt (0.5% NaCl, NS) diet, high-salt (8% NaCl, HS) diet, or high-salt diet plus BPS treatment (HS-BPS) for 3 weeks. All rats were randomly assigned to NS, HS, or HS-BPS group.

**Figure 1:**
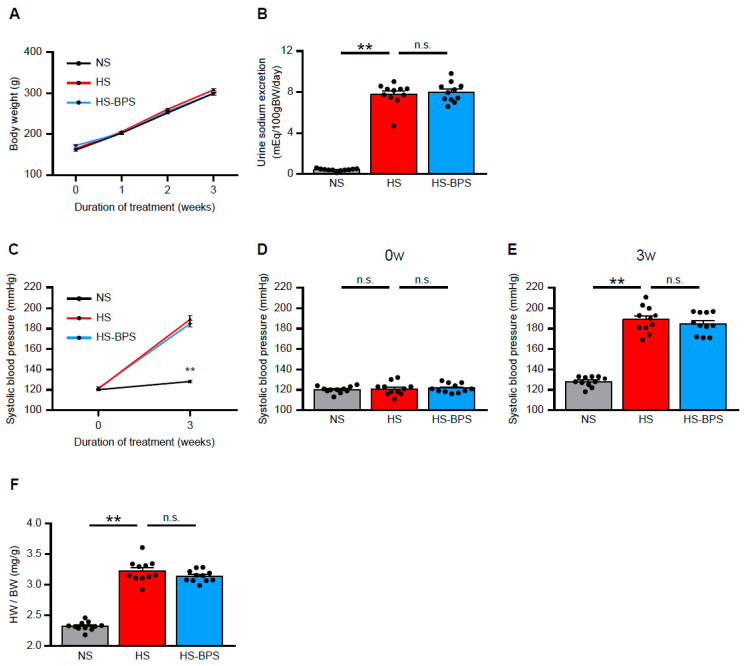
Effects of BPS on blood pressure levels in high-salt-fed DS rats. (**A**) Change in body weight, (**B**) urine sodium excretion levels at 3 weeks, (**C**–**E**) systolic blood pressure levels at 0 and 3 weeks, and (**F**) heart weight to body weight ratio of DS rats on a normal-salt (0.5% NaCl, NS) diet, high-salt (8% NaCl, HS) diet or high-salt diet plus BPS treatment (HS-BPS) for 3 weeks (*n* = 11 per group). The asterisks in (**C**) correspond to NS vs. HS group at 3 weeks. Data are expressed as mean ± SEM. ** *p* < 0.01; n.s., not significant.

There were no significant changes in body weight after high-salt feeding with or without BPS treatment (Figure 1A). High-salt feeding increased urine sodium excretion at 3 weeks; however, BPS did not have any effect on sodium excretion (Figure 1B). Systolic blood pressure levels were similar among NS, HS, and HS-BPS rats at baseline (Figure 1C,D). BPS treatment slightly, but insignificantly, decreased blood pressure levels (Figure 1C,E). Heart weight to body weight ratios, the marker of cardiac hypertrophy, were higher in HS rats compared with NS rats (Figure 1F). These ratios remained unchanged by BPS treatment (Figure 1F).

### 2.2. Administration of BPS Ameliorates Proteinuria and Renal Injury in High-Salt-Fed DS Rats

**Figure 2:**
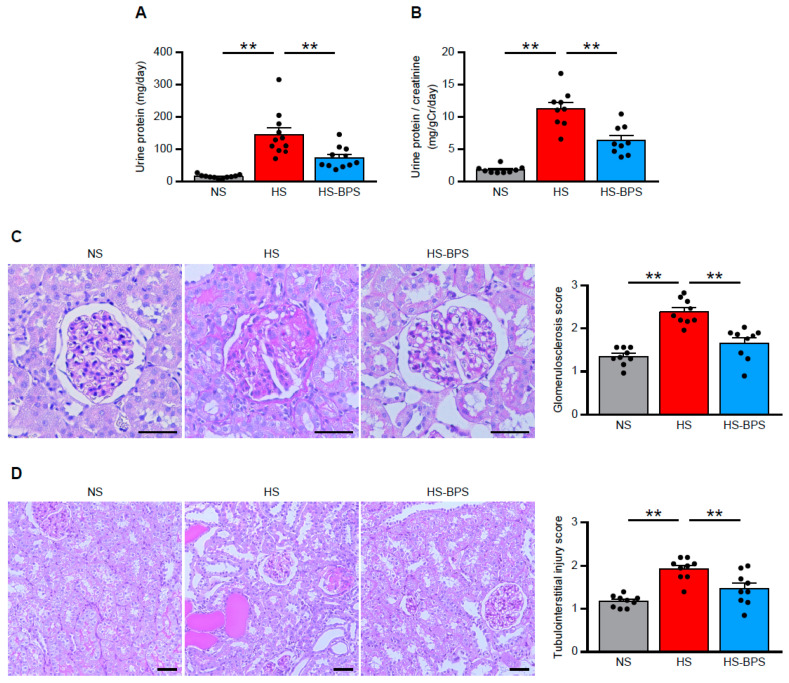
BPS administration ameliorates proteinuria and renal injury in high-salt-fed DS rats. (**A**) Levels of urine protein excretion at 3 weeks and (**B**) urine protein per creatinine excretion in NS, HS, and HS-BPS rats (*n* = 11 per group). (**C**) Representative photomicrographs of period acid–Schiff (PAS)-stained kidney sections and quantification of glomerulosclerosis (see also Methods; *n* = 9 per group). Scale bars, 50 μm. (**D**) Representative photomicrographs of PAS-stained kidney sections and quantification of tubulointerstitial injury (see also Methods; *n* = 9 per group). Scale bars, 50 μm. Data are expressed as mean ± SEM. ** *p* < 0.01.

We measured the urine protein excretion levels at 3 weeks to evaluate whether BPS exerted protective effects on renal injury in HS rats. HS rats showed the overt increase in urine protein excretion (Figure 2A,B). Of interest, urine protein excretion was markedly reduced by BPS treatment (Figure 2A,B).

To determine whether BPS ameliorated renal injury, we also addressed renal histology in periodic acid–Schiff (PAS)-stained kidney sections. HS rats displayed glomerulosclerosis and tubulointerstitial injury manifested by vacuolation and desquamation of the renal epithelial cells, accompanied by proteinaceous cast formation (Figure 2C,D), in agreement with previous reports [7,25]. Our preliminary results showed that there was very little fibrosis in HS rats, indicating that HS rats have salt-induced kidney injury in early stage. Semiquantitative evaluation of renal histology demonstrated that BPS significantly attenuated glomerular and tubulointerstitial damages (Figure 2C,D). Renal histology also revealed the strong correlation between glomerular and tubulointerstitial damages in HS and HS-BPS rats (*r* = 0.80, *p* < 0.01) (Supplementary Figure S1A), indicating that BPS treatment attenuated tubulointerstitial damages along with the mitigation of glomerular damages. Moreover, BPS significantly reduced serum creatinine levels in high-salt-fed DS rats (Supplementary Figure S1B). BPS also decreased blood urea nitrogen (BUN) levels, however, the decrease was not statistically significant (HS: 24.4 ± 0.9 vs. HS-BPS: 22.4 ± 0.5 mg/dL; *p* = 0.065) (Supplementary Figure S1C).

### 2.3. BPS Reduced Renal MR Pathway Activation via Rac1 Activity Suppression in High-Salt-Fed DS Rats

We next explored the possible mechanism of the renoprotective effects observed in HS-BPS rats. Accumulating evidence suggests that MR and its ligand, aldosterone, play a pivotal role in the progression of kidney injury [26,27,28], including salt-induced kidney injury [7,25,29].

**Figure 3:**
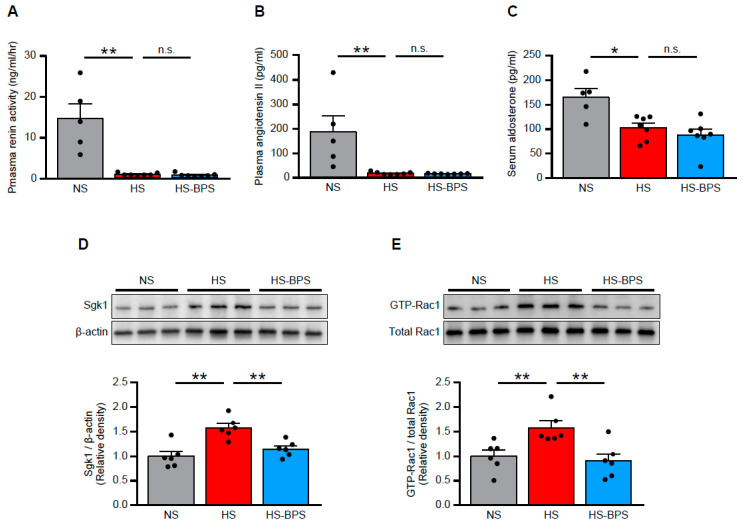
BPS reduced renal MR pathway activation through Rac1 activity suppression in high-salt-fed DS rats. (**A**) Plasma renin activity, (**B**) plasma angiotensin II concentration, and (**C**) serum aldosterone concentration in NS, HS, and HS-BPS rats (*n* = 5 for NS, *n* = 7 for HS, *n* = 7 for HS-BPS rats). (**D**) Western blot analysis of MR downstream effector Sgk1 in the renal cortex. Blots show biological replicates, and bar graphs show the results of quantitation (*n* = 6 per group). (**E**) Western blot analysis of GTP-bound Rac1 in the renal cortex. Blots show biological replicates, and bar graphs show quantitation results (*n* = 6 per group). Data are expressed as mean ± SEM. * *p* < 0.05; ** *p* < 0.01; n.s., not significant.

Thus, we evaluated renin-angiotensin-aldosterone system (RAAS) in our animal models. High-salt feeding suppressed plasma renin activity, angiotensin II concentrations, and serum aldosterone concentrations (Figure 3A–C), indicating that RAAS was suppressed by high-salt feeding. In spite of suppressed aldosterone levels, HS rats demonstrated increased expressions of Sgk1 (Figure 3D), a downstream molecule of MR signaling, indicating MR activation in HS rats.

With regard to the mechanism of salt-induced MR activation, we previously identified the role of Rac1 as a modulator of MR activity [6,7,8]. We investigated Rac1 activity in the renal cortex of HS rats to confirm whether renal Rac1 is activated. HS rats showed increased expressions of GTP-Rac1 (Figure 3E), an active form of Rac1, consistent with previous findings [7].

BPS treatment suppressed MR signaling as assessed by reduced Sgk1 expressions (Figure 3D), despite unchanged serum aldosterone levels (Figure 3C), indicating that MR signaling was ligand independently regulated. In addition, renal Rac1 activity was suppressed by BPS administration. These results suggested that BPS reduced MR overactivation through renal Rac1 activity suppression.

### 2.4. BPS Alleviates Renal Inflammation in High-Salt-Fed DS Rats

**Figure 4:**
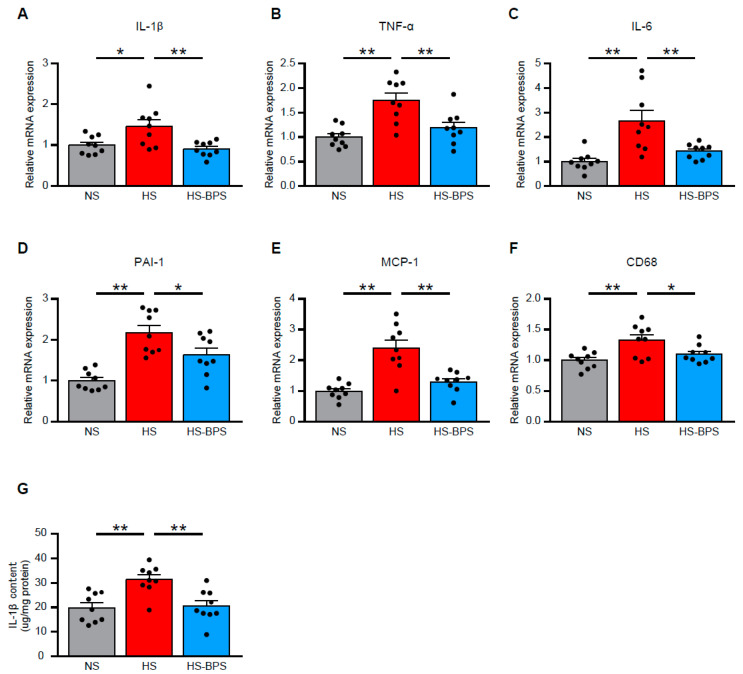
BPS alleviates renal inflammation in high-salt-fed DS rats. Quantitative analysis of (**A**) *IL-1β*, (**B**) *TNF-α*, (**C**) *IL-6*, (**D**) *PAI-1/SERPINE1*, (**E**) *MCP-1/CCL2*, and (**F**) *CD68* gene expression in the renal cortex by real-time RT-PCR (*n* = 9 per group). (**G**) IL-1β content in renal cortex evaluated by ELISA (*n* = 9 per group). Data are expressed as mean ± SEM. * *p* < 0.05; ** *p* < 0.01.

We next investigated the factors inducing renal Rac1 activation in our model. Previous studies documented the role of inflammation that is responsible for Rac1 activation [21,22,23]. Therefore, we quantitatively analyzed the gene expression of several cytokines. Quantitative real-time RT-PCR revealed that the expression of pro-inflammatory cytokines including interleukin-1β (*IL-1β*), tumor necrosis factor-α (*TNF-α*), interleukin-6 (*IL-6*), plasminogen activator inhibitor-1 (*PAI-1/SERPINE1*), and monocyte chemoattractant protein-1 (*MCP-1/CCL2*) were significantly increased by high-salt feeding (Figure 4A–E). Moreover, HS rats also showed increased expression of *CD68* (Figure 4F), a marker of macrophage expression. We measured IL-1β in proteins of renal cortex by enzyme-linked immunosorbent assay (ELISA) to validate the gene expression. We found elevated IL-1β levels in renal proteins of HS rats (Figure 4G). BPS administration significantly reduced all of these pro-inflammatory cytokine gene expressions and IL-1β protein expressions (Figure 4A–G), indicating that inflammation in the kidney of high-salt-fed DS rats was alleviated by BPS treatment.

## 3. Discussion

**Figure 5:**
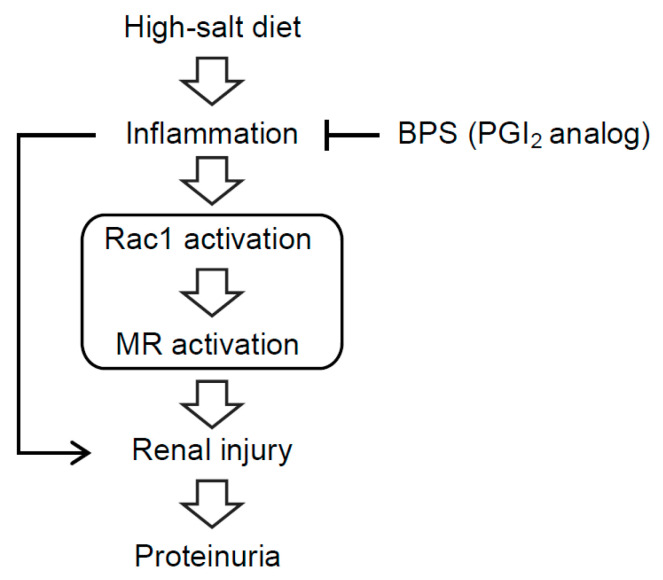
Schematic diagram illustrating the hypothesis that Rac1-MR pathway activation by inflammation is involved in the progression of renal injury in high-salt-fed DS rats. In high-salt-fed DS rats, inflammation-induced Rac1-MR pathway activation is involved in the progression of renal injury and massive proteinuria. Treatment of BPS, a PGI_2_ analog, suppresses inflammation, leading to the reduction of Rac1-MR activation and kidney damage.

Using DS rats as a model of salt-sensitive hypertension, this study demonstrated that BPS counteracts against the progression of salt-induced kidney injury. High-salt diet induced overt proteinuria, accompanied by glomerulosclerosis and tubulointerstitial injury in DS rats. High-salt-fed DS rats showed increased expressions of active Rac1 and Sgk1, a downstream molecule of MR signaling, indicating Rac1-MR pathway activation, associated with elevated renal inflammation. BPS administration attenuated proteinuria and renal injury. Moreover, Rac1-MR activation and inflammation in the kidney of high-salt-fed DS rats were ameliorated by BPS treatment, which suggested that inflammation-induced Rac1-MR pathway activation is involved in the progression of salt-induced kidney injury and that BPS treatment suppresses renal inflammation, leading to the attenuation of Rac1-MR activation and kidney damage (Figure 5).

In this study, we used DS rats that serve as an excellent model of salt-sensitive hypertension and associated kidney injury, exhibiting many phenotypic characteristics common in human hypertension [24]. Increasing evidence suggests the role of inflammation in the progression of chronic kidney disease [30,31]. Furthermore, salt-sensitive hypertension in human and experimental animal models has been demonstrated to be accompanied by progressive kidney damage leading to end-stage renal disease, which is associated with elevated inflammation [1,2,3].

We speculate that there are several possibilities explaining the mechanisms whereby BPS treatment had renoprotective effects in this model. First, BPS can exert anti-inflammatory effects in salt-induced kidney injury. Accumulating evidence shows that BPS has renoprotective effects in various experimental kidney disease models [11,12,13,14,15,16,17,19]. Indeed, the observed renal protective effects of BPS in this study were associated with the reduction of inflammation. These data are in line with previous reports showing anti-inflammatory roles of BPS [11,14,16,18].

A second possibility is that BPS treatment reduced renal injury through the suppression of Rac1-MR pathway activation in the kidney. We have demonstrated that salt loading increases Rac1 activity in the kidneys of DS rats, which is associated with MR activation, resulting in salt-induced kidney injury [7]. However, in this previous study, it remained undetermined what induced Rac1 activation. Many investigators have evaluated factors modulating Rac1 activity. Some studies reported that Rac1 can be activated by inflammatory cytokines [21,22,23], dietary salt [6,7], angiotensin II [32,33], angiotensin II with salt [34], aldosterone [26], high glucose [35], reactive oxygen species [36], and mechanical stress [37]. Of note, the anti-inflammatory effect of BPS was prominent in this study. Therefore, it is possible that BPS suppressed renal Rac1-MR activation through the reduction of inflammation in the kidney.

Third, BPS might decrease blood pressure. BPS has been used for the treatment of pulmonary arterial hypertension [9] and peripheral arterial disease [10]. Although BPS has a vasodilatory effect, it is not an antihypertensive drug. Indeed, hypertension is a major risk factor for chronic kidney disease progression, which can also occur as a consequence of a progressive decline in renal function [38]. In the present study, three weeks of BPS treatment slightly, but insignificantly, decreased blood pressure by tail-cuff method. In the further study, it is important to evaluate continuous blood pressure measured by radiotelemetry.

The Rho GTPases (Rac1, RhoA, and Cdc42) act as molecular switches that regulate actin dynamics [39], and consequently play a pivotal role in maintaining the cytoskeletal and molecular integrity of the podocyte foot processes in a dynamic manner [40]. Changes of activity of these GTPases lead to hypo- or hyper-motility of podocytes, resulting in proteinuria [40,41]. Therefore, Rho GTPases have drawn attention as potential therapeutic applications in CKD. We previously demonstrated that high-salt feeding induced hypertension and podocyte injury in DS rats [25]. In this study, eplerenone, a MR antagonist, and hydralazine reduced blood pressure to the similar extent in high-salt-fed DS rats; however, podocyte injury and urine protein excretion were ameliorated only in eplerenone-treated group [25]. These findings raised the possibility that the reduction of urine protein by BPS treatment in the current study was mediated via the suppression of Rac1-MR pathway activation in podocytes.

With respect to the clinical application of BPS for the treatment of moderate to severe CKD patients, phase II clinical trial showed that TRK-100 STP (sustained-release BPS) significantly prevented the estimated glomerular filtration rate reduction in patients with non-diabetic CKD patients [42], but Phase III (CASSIOPEIR) trial did not prove a beneficial effect in CKD progression [43]. Another phase III trial of aldosterone antagonist, spironolactone, in patients with preserved cardiac function heart failure (TOPCAT) did not achieve a significant reduction in the primary composite outcome of death from cardiovascular causes, aborted cardiac arrest, or hospitalization for the management of heart failure [44]. However, in a sub-analysis of this trial, an unusually large difference was identified in the placebo event rates between the sites conducting TOPCAT in the four countries in America compared with those in Russia and Georgia [45]. To explain the regional difference, investigators measured metabolite concentrations of spironolactone and found significant regional discrepancies in the reported use and the actual use of spironolactone [46]. These results imply that medication adherence is important to achieve therapeutic goals, and that a sub-analysis of CASSIOPEIR trial according to BPS itself or metabolite of BPS concentrations is needed. Moreover, a sub-analysis study to estimate dietary salt intake in individual patients is also needed, since high salt activate Rac1-MR pathway in hypertensive CKD patients [47].

Prostaglandins appear when arachidonic acid is released from the plasma membrane by phospholipases and metabolized by the peroxidase actions of cyclooxygenases to prostaglandin H_2_, which can be thereafter converted into more stable biologically active prostaglandins, including PGI_2_ and prostaglandin E_2_ (PGE_2_) [48,49]. Prostaglandins exert various functions in the pathology and physiology of kidney, in which the levels of prostaglandins can be regulated at multiple steps [48,49]. Several investigators reported that inhibition of prostaglandin production with either nonselective or selective inhibitors of cyclooxygenase-2 (COX-2) activity can induce or exacerbate salt-sensitive hypertension [50,51,52]. Notably, the protective effect of PGI_2_ activity against the development of atherothrombotic cardiovascular disease has been demonstrated to be mediated by the inhibition of various cellular processes, including platelet activation, leukocyte adhesion, as well as vascular smooth muscle cell modulation [53]. Consistently, salt-loaded DS rats showed lower urine excretion of 6-keto PGF1α, a metabolite of PGI_2_, as compared to salt-loaded DR rats [54], suggesting that decreased production of PGI_2_ in the kidney contribute to the development of salt-sensitive hypertension, although we did not measure these metabolites in this study. With respect to the relationship between prostaglandins and salt-sensitive hypertension, moreover, a recent study reveals that COX-2 derived PGE_2_ in macrophages plays an important role in both kidney and skin in maintaining homeostasis in response to chronically increased dietary salt [55]. Therefore, this study also implies that inhibiting COX-2 expression or activity in macrophages can result in a predisposition to salt-sensitive hypertension [55]. Thus, it is possible that prostaglandins in macrophages play roles in the development of salt-sensitive hypertension and subsequent salt-induced renal injury in our model, which is a subject for future study.

A limitation of our study is that the exact mechanisms whereby BPS suppresses Rac1-MR activation remain undetermined. Direct demonstration by cell culture experiments are needed to clarify the mechanisms, which was not addressed in this study. It is also unknown whether BPS elicits primary protective effects in the kidney. Given that the renoprotective effect of BPS was evaluated only in male rats, the result may not apply to female rats. Despite these limitations, this study clearly demonstrates that BPS has renoprotective effects in salt-induced kidney injury. Given our data, we speculate that anti-inflammatory effects of BPS reduced Rac1-MR activation in the kidney, leading to the attenuation of renal damage. Our data indicate that BPS can be a therapeutic option to treat CKD, especially in patients with early stage of CKD. Additional prospective clinical studies are needed to further address this hypothesis.

## 4. Materials and Methods

### 4.1. Animals and Experimental Design

Animal care and treatment complied with the standards described in the Guidelines for the Care and Use of Laboratory Animals of the University of Tokyo (Tokyo, Japan). All studies were approved by the Institutional Animal Care and Use Committee of the University of Tokyo (RAC 140202, date: 1 July 2014). Five-week-old male DS rats were purchased from Japan SLC (Shizuoka, Japan), which were randomly divided into three groups as follows: a normal-salt (0.5% NaCl) diet group (NS, *n* = 11), a high-salt (8% NaCl) diet group (HS, *n* = 11), and a high-salt diet plus BPS (provided by Toray Industries, Inc., Tokyo, Japan, 750 mg/kg/day in drinking water), a PGI_2_ analog, treatment group (HS-BPS, *n* = 11). The dosages of BPS used were determined according to the renoprotective effects in previous studies [12,19]. The rats were fed either a normal-salt (MF diet, Oriental Yeast, Tokyo, Japan) or a high-salt (MF diet containing 8% NaCl, Oriental Yeast) diet for 3 weeks. The NS group was used as control. Body weight was recorded every week during the experimental period. All rats had free access to drinking water and food under temperature-controlled conditions and a 12-h light/dark cycle.

### 4.2. Blood Pressure Measurements in Conscious Rats

The systolic blood pressures of conscious rats were measured at 3 weeks using the tail-cuff method (BP-98A; Softron, Tokyo, Japan), as previously described [7,56]. Measurements were performed at daytime and the average of five serial measurements taken in a calm state was calculated for each rat. A dark cover was placed over animals to reduce stress.

### 4.3. Metabolic Studies

Twenty-four-hour urine samples were collected using an individual metabolic cage (Natsume, Tokyo, Japan) at 3 weeks. Urine protein and creatinine levels were measured at SRL (Tokyo, Japan).

### 4.4. Blood Collection and Laboratory Measurements

At the end of the experiments, we extracted blood from the inferior vena cava with syringes under pentobarbital anesthesia. Blood was immediately transferred into a blood collection tube containing EDTA-2Na and centrifuged at 5000 rpm for 20 min at 4 °C; then plasma was removed. The remaining blood volume was transferred into a blood collection tube containing serum-separating medium and centrifuged at 5000 rpm for 20 min at 4 °C; then serum was removed. Plasma and serum were stored at −30 °C until analyses. Plasma renin activity, plasma angiotensin II concentrations, and serum aldosterone concentrations were determined by radioimmunoassay (SRL). Serum creatinine and blood urea nitrogen levels were measured at SRL.

### 4.5. Renal Histology

Kidneys were harvested and fixed in 4% paraformaldehyde overnight at 4 °C and embedded in paraffin. Subsequently, 3-μm-thick tissue sections were stained with PAS reagent. Pathological changes in kidney tissues were observed under a light microscope (Leica DMI 4000B, Leica Microsystems, Wetzlar, Germany). We semiquantitatively assessed the degrees of glomerular damage and tubulointerstitial injury according to an established scoring system [6,7,26]. For evaluation of glomerulosclerosis, glomeruli were scored on a scale of 0 to 4, according to the following criteria: 0 normal; 1, involvement of 1–25% of the glomerular tufts; 2 involvement of 26–50% of the glomerular tufts; 3, involvement of 51–75% of the glomerular tufts; and 4, involvement of 75–100% of the glomerular tufts. Fifteen glomeruli (×200) of each kidney were scored and the mean value was calculated as the glomerulosclerosis score. Tubulointerstitial injury was defined as tubular cast formation, tubular dilatation, tubular atrophy, or inflammatory cell infiltration. The extent of damage was scored 0 to 4, according to the following criteria: 0, normal; 1, involvement of 1–25% of the cortex; 2, involvement of 26–50% of the cortex; 3, involvement of 51–75% of the cortex; and 4, involvement of 75–100% of the cortex. Ten cortical fields (×100) of each kidney were scored and the mean value was defined as the tubulointerstitial injury score. A blind evaluation of the glomerulosclerosis and tubulointerstitial injury was done by two renal pathologists.

### 4.6. Western Blot Analysis

Kidneys were removed, snap frozen in liquid nitrogen, and stored at −80 °C until homogenization. Kidneys were homogenized on ice with extraction buffer containing 50 mM Tris-HCl (pH 8.0), 150 mM NaCl, 1% NP-40, 0.5% sodium deoxycholate, 0.1% SDS, and protease inhibitors (Complete, Roche Diagnostics, Basel, Switzerland) and were centrifuged at 14000 rpm at 4 °C for 30 min to obtain the cellular proteins in the supernatant. Protein concentrations were determined using a BCA protein assay (Fujifilm Wako Pure Chemical Corporation, Osaka, Japan).

Western blotting (WB) was performed as previously described [57], with some modifications. Briefly, equal amounts of protein were mixed with 2× Laemmli sample buffer, boiled for 10 min, separated on polyacrylamide gels, and transferred to polyvinylidene fluoride (PVDF) membranes. The membranes were blocked with PVDF-blocking reagent (Toyobo, Osaka, Japan) for 30 min at room temperature and incubated with primary and peroxidase-conjugated secondary antibodies. Signals were detected using the ECL Prime Western Blotting Detection Reagent (GE Healthcare, Waukesha, WI, USA) and scanned using ImageQuant LAS 4000 mini (GE Healthcare). Signal intensity was quantitated using ImageJ 1.46r software (National Institutes of Health, Bethesda, MD, USA).

### 4.7. Quantitative RT-PCR Analysis

Total RNA was isolated from kidneys using an RNeasy mini kit (Qiagen, Venlo, Netherlands) according to the manufacturer’s instructions. RNA quantity and purity were assessed via Nanodrop 2000 spectrophotometer (Thermo Scientific, Waltham, MA, USA). Total RNA having OD 260/280 ratio between 2.0 and 2.2 was used for reverse transcription. cDNA was synthesized using RevatraAce reverse transcriptase (Toyobo) according to the manufacturer’s instructions. Quantitative RT-PCR analyses were performed using a StepOnePlus Real-Time PCR System (Applied Biosystems, Waltham, MA, USA), as described previously [58]. The expression levels of *TNF-α*, *PAI-1/SERPINE1*, *MCP-1/CCL2*, and glyceraldehyde 3-phosphate dehydrogenase (*GAPDH*) were then analyzed using the TaqMan Universal PCR Master Mix (Applied Biosystems). TaqMan primer/probes sets (Applied Biosystems) were used, and the GenBank accession number and assay ID are as follow: *TNF-α* (NM_012675.3, RN01525859_g1), *PAI-1/SERPINE1* (NM_012620.1, RN01481341_m1), *MCP-1/CCL2* (NM_031530.1, RN00580555_m1), and *GAPDH* (NM_017008.4, Rn01775763_g1). SYBR Green PCR Master Mix (Applied Biosystems) was used with primers for detection of other genes (Supplementary Table S1). Denaturation took place at 95 °C for 15 s and annealing and extension at 60 °C for 1 min for 40 cycles. The comparative cycle threshold method was used to compare gene expression levels. Expression levels were normalized against the housekeeping gene *GAPDH*.

### 4.8. IL-1β ELISA

Renal IL-1β protein concentrations were determined by enzyme-linked immunosorbent assay (rat IL-1β/IL-1F2 Quantikine ELISA, RLB00, R&D Systems, Minneapolis, MN, USA). Kidney was completely homogenized in an extraction buffer containing 10 mM Tris-HCl (pH 7.8), 150 mM NaCl, 1% NP40, 1 mM EDTA, and protease inhibitors (Complete, Roche Diagnostics). After homogenization, samples were centrifuged at 4 °C, 14,000 rpm for 30 min. The clean supernatant was then used to determine IL-1β protein concentration, according to the manufacturer’s protocol.

### 4.9. Antibodies

Primary antibodies used in WB studies included those for Sgk1 (SAB2104902, 1:6000; Sigma-Aldrich, St. Louis, MO, USA), β-actin (ab8227, 1:5000; Abcam, Cambridge, UK), GTP-Rac1 (#26903, 1:3000; NewEast Biosciences, Malvern, PA, USA), and total Rac1 (#05-389, 1:6000; Millipore, Billerica, MA, USA).

### 4.10. Statistical Analyses

Data are expressed as mean ± SE. For comparison across multiple groups, one-way or two-way ANOVA followed by a Tukey–Kramer post hoc test was performed. *p* < 0.05 was considered statistically significant. Statistical analyses were performed with GraphPad Prism 7 (GraphPad Software, La Jolla, CA, USA).

## Supplementary Materials

The following are available online at https://www.mdpi.com/1422-0067/21/12/4433/s1.

## Abbreviations

BPS	beraprost sodium
CKD	chronic kidney disease
DR rats	Dahl salt-resistant rats
DS rats	Dahl salt-sensitive rats
ELISA	enzyme-linked immunosorbent assay
GBM	glomerular basement membrane
IL-1β	interleukin-1β
IL-6	interleukin-6
MCP-1	monocyte chemoattractant protein-1
MR	mineralocorticoid receptor
PAI-1	plasminogen activator inhibitor-1
PAS	periodic acid–Schiff
RAAS	renin-angiotensin-aldosterone system
Sgk1	serum and glucocorticoid-regulated kinase
TNF-α	tumor necrosis factor-α
