# Supplementary material for: PGI2 Analog Attenuates Salt-Induced Renal Injury through the Inhibition of Inflammation and Rac1-MR Activation
Source: Int J Mol Sci. 2020 Jun 22;21(12):4433. doi: 10.3390/ijms21124433 (PMC7353033; doi:10.3390/ijms21124433)
Supplement: Supplementary file 1 [file ijms-21-04433-s001.pdf]

Supplementary Materials

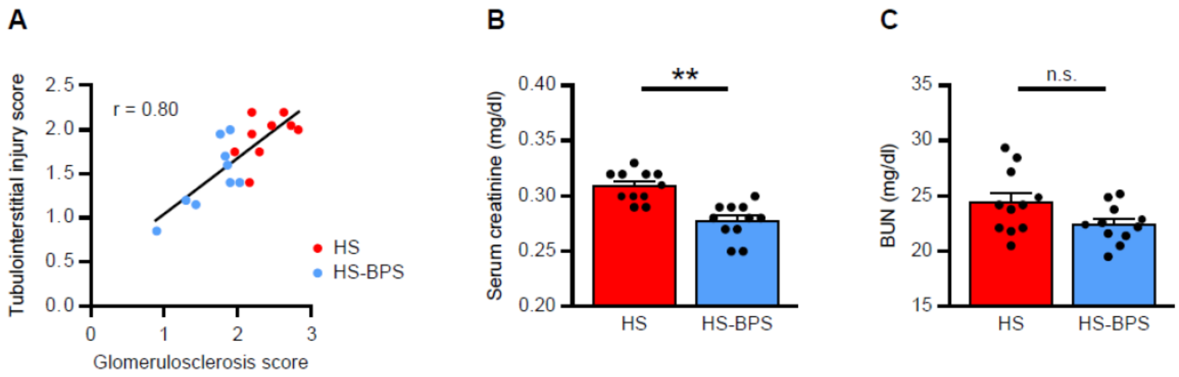

Figure S1. Relationship between glomerulosclerosis and tubulointerstitial scores, serum creatinine and BUN levels in high-salt-fed DS rats with or without BPS treatment. (**A**) Relationship between glomerulosclerosis and tubulointerstitial scores in HS and HS-BPS rats ($n = 9$ per group). (**B**) Serum creatinine and (**C**) BUN levels in HS and HS-BPS rats ($n = 11$ per group). Data are expressed as mean ± SEM. ** $p < 0.01$; n.s., not significant.

Table S1. List of primer pairs used in this study.

mRNA	Accession Number	Primer Pair (5′–3′)	
IL-1β	NM_031512.2	Forward	AAAAATGCCTCGTGCTGTCT
		Reverse	CAGGGATTTTGTCGTTGCTT
IL-6	NM_012589.2	Forward	AGAGACTTCCAGCCAGTTGC
		Reverse	TGAAGTAGGGAAGGCAGTGG
CD68	NM_001031638.1	Forward	CAAAAAGGCTGCCACTCTTC
		Reverse	CTTCCGGTGGTTGTAGGTGT
